# Supplementary material for: Use of multifocal electroretinograms to determine stage of glaucoma
Source: PLoS One. 2023 Jan 12;18(1):e0278234. doi: 10.1371/journal.pone.0278234 (PMC9836278; doi:10.1371/journal.pone.0278234)
Supplement: Attachment — Submitted filename: Response to Reviewers .docx [file pone.0278234.s003.docx]

We thank the editor and reviewers for their suggestions and comments. These were helpful in allowing us to revise our manuscript to make it better. We have written the revised parts with red fonts in the text and addressed all of the comments below.

I have also edited the Method section to include ethics statement as it was pointed out by the editor. It is also in red fonts.

Reviewer #1: The authors investigated the role of multifocal electroretinograms to determine stage of glaucoma. They used the amplitudes of the positive peaks (P1, P2) of the second order kernels in the nasal and temporal fields within central 7.5° diameter, reporting that the parameters were significantly different among normal and glaucoma, and glaucoma groups classified by glaucoma severity. Although their study is interesting, it is necessary to suggest the sufficient rationale for assessing nasal side of the macular lesion and nasal to temporal asymmetry in glaucoma.

Glaucomatous damage is characterized by the superior and inferior asymmetry, not nasal and temporal asymmetry, and most frequently affected lesion of glaucoma at the early stage of disease is the temporal side (nasal step defect in early glaucoma.

**Answer:** We have added the rationale for assessing the nasal side of the macular lesion and nasal to temporal asymmetry in glaucoma in the Introduction section.

And we have added the report of Lee et al in the References.

**Reviewer #2:**

This is an interesting study evaluation of the " To determine whether multifocal electroretinograms (mfERGs) recorded with natural pupils and skin electrodes can be used to assess the stage of open angle glaucoma (OAG). "The scientific work done commendable. Overall manuscript is well-written and easy to read with good explanations and interesting findings.

1- There are so many grammatical and syntax errors that need to be addressed.

**Answer:** This revised copy was edited by native English speaking Professor Emeritus Duco Hamasaki of the Bascom Palmer Eye Institute.

2- However, the pattern ERGs (pERGs) have been shown to managing glaucoma.” The sentence is vague and needs to be rephrased.”

**Answer:** We have revised this sentence in the Introduction section.

3- “we recorded mfERGs with natural pupil using skin electrodes, and we focused on the nasal responses within the central 15º.” It is better to write why you focus on nasal responses in the introduction too.

**Answer:** We have added the reason for why we focused on the nasal responses in the Introduction section (same as Reviewer #1).

4- The mfERG results may be influenced by age, how did the authors consider the effect of age on the mfERG results. Are the groups matched based on age? As you have mentioned, patients with moderate and advanced glaucoma were also significantly older than the normal subjects

**Answer:** We have described the effects of age on P23 *(revised manuscript with track changes)*, and we have added paper by Nabeshima T in the References.

5- The authors conclude that “These results indicate that the amplitudes of P1 and P2 in the nasal visual field of the second-order response of the mfERG are a good marker for the stage of glaucoma.” It is better to compare the mentioned parameters (P1 amplitude,…) between the groups. (between preperimetric and mild, mild and moderate, …)

**Answer:** We compared the parameters between the groups and described the results in the Results section and Table 3 (new Table).

6- Although the authors showed a significant correlation between the mfERG parameters and MD, cpRNFL, and mRNFL but I can not find any correlation between the mf ERG parameters and the glaucoma stage.

**Answer:** We have compared the parameters between the groups and listed the Results in Table 3.

On p19-20 *(revised manuscript with track changes)* of the Discussion section, we also described these findings in the Results section.

7- Please summarize the result section, you can omit the parts which have been mentioned in table 1.

**Answer:** We have deleted the parts which have been listed in Table 1.

8- Please explain the p values in table 2

**Answer:** We have added an explanation of the *P* values.
